# Effect of Equal Daily Doses Achieved by Different Power Densities of Low-Level Laser Therapy at 635 nm on Open Skin Wound Healing in Normal and Diabetic Rats

**DOI:** 10.1155/2014/269253

**Published:** 2014-01-16

**Authors:** Róbert Kilík, Lucia Lakyová, Ján Sabo, Peter Kruzliak, Kamila Lacjaková, Tomáš Vasilenko, Martina Vidová, František Longauer, Jozef Radoňak

**Affiliations:** ^1^1st Department of Surgery, Pavol Jozef Šafárik University, Medical Faculty and Louis Pasteur University Hospital, Trieda SNP1, 040 11 Košice, Slovakia; ^2^Department of Medical and Clinical Biophysics, Pavol Jozef Šafárik University, Medical Faculty, Košice, Slovakia; ^3^Department of Cardiovascular Diseases, International Clinical Research Center, St. Anne's University Hospital and Masaryk University, Pekarska 53, 656 91 Brno, Czech Republic; ^4^1st Department of Internal Medicine, Pavol Jozef Šafárik University, Medical Faculty and Louis Pasteur University Hospital, Košice, Slovakia; ^5^Department of Surgery, Pavol Jozef Šafárik University, Medical Faculty and 1st Private Hospital, Košice-Šaca a.s., Košice, Slovakia; ^6^Department of Forensic Medicine, Pavol Jozef Šafárik University, Medical Faculty, Košice, Slovakia

## Abstract

*Background and Objective. *Despite the fact that the molecular mechanism of low-level laser therapy (LLLT) is not yet known, the exploitation of phototherapy in clinical medicine and surgery is of great interest. The present study investigates the effects of LLLT on open skin wound healing in normal and diabetic rats. *Materials and Methods.* Four round full-thickness skin wounds on dorsum were performed in male adult nondiabetic (*n* = 24) and diabetic (*n* = 24) Sprague–Dawley rats. AlGaInP (635 nm, wavelength; 5 J/cm^2^, daily dose) was used to deliver power densities of 1, 5, and 15 mW/cm^2^ three times daily until euthanasia. *Results.* PMNL infiltration was lower in the irradiated groups (15 mW/cm^2^). The synthesis and organisation of collagen fibres were consecutively enhanced in the 5 mW/cm^2^ and 15 mW/cm^2^ groups compared to the others in nondiabetic rats. In the diabetic group the only significant difference was recorded in the ratio PMNL/Ma at 15 mW/cm^2^. A significant difference in the number of newly formed capillaries in the irradiated group (5, 15 mW/cm^2^) was recorded on day six after injury compared to the control group. *Conclusion.* LLLT confers a protective effect against excessive inflammatory tissue response; it stimulates neovascularization and the early formation of collagen fibres.

## 1. Introduction

Wound healing consists of three phases—inflammation, tissue formation, and tissue remodeling—which overlap in time. It is a dynamic, interactive process involving soluble mediators, blood cells, extracellular matrix, and parenchymal cells [1]. Impaired wound healing is still a significant problem in clinical practice. One of the leading causes of impaired wound healing is diabetes mellitus. There are many factors that contribute to the altered tissue repair of diabetes mellitus, but the exact pathogenesis of poor wound healing is not completely understood [2–4]. Evidence from studies involving human and animal models of diabetes reveals several abnormalities in the various phases of the wound healing process [4, 5]. Poor wound healing with diabetes mellitus has been shown to be associated with hyperglycemia, inhibition of inflammatory response, poor angiogenesis, and fibroplasia and defects in collagen deposition and differentiation of the extracellular matrix [5–8]. The causes of the impaired resistance to infection are multifactorial. Hyperglycemia may increase available nutrients for bacteria and may also impair local defenses. Leukocytes have different forms of impaired function in a hyperglycemic environment. In addition, other factors contribute to impaired immune function in diabetes mellitus [2]. Delayed wound healing is still a significant problem in clinical practice in general, despite the use of many promising physical methods, such as vacuum assisted closure (VAC) [9, 10], light emitting diodes (LEDs) [11], and low-level laser therapy (LLLT) [12, 13].

LLLT belongs to a group of modern experimental approaches used in wound healing therapy [14]. LLLT as a therapeutic modality was introduced by the work of Mester and colleagues, who noted an improvement in wound healing with the application of a low-energy (1 J/cm^2^) ruby laser [13, 15]. It had been well documented previously that red lasers reduce pain and inflammation, increase collagen deposition, and accelerate wound closure during wound healing. On the other hand, some studies have shown that LLLT may be ineffective [16, 17]. One of the reasons for its ineffectiveness may be associated with the use of extremely low doses [14, 18].

The exact mechanism of action of LLLT in wound healing is still not fully clarified. Optimal parameters of LLLT are still not defined. Therefore, the aim of our study was to compare the influence of different power densities, achieving equal daily doses (5 J/cm^2^), on skin wound healing in nondiabetic laser-treated and diabetic laser-treated rats, using an excisional model and histological evaluation.

## 2. Materials and Methods

### 2.1. Animals

Ten-month-old male Sprague-Dawley rats (*n* = 48), weighing 500–550 g, were included in the experiment and randomly divided into two groups of 24 animals, that is, nondiabetic laser-treated group (N) and diabetic laser-treated group (D). This experiment was approved by the Ethics Committee of the Faculty of Medicine of P. J. Šafárik University and by the State Veterinary Administration of the Slovak Republic.

#### 2.1.1. Animal Model

Six weeks prior to the wound healing experiment animals received 60 mg/kg of streptozotocin (Streptozotocin, Sigma-Aldrich, Prague, Czech Republic). For intraperitoneal administration 15 mg of streptozotocin was prepared in 1 mL phosphate buffer (pH = 5.5). Rats with glycemia higher than 12 mmol/L (over three consecutive days) were included in the experiment.

In general anesthesia induced by intramuscular administration of ketamine (40 mg/kg; Narkamon a.u.v., Spofa a.s., Prague, Czech Republic), xylazine (11 mg/kg; Rometar a.u.v., Spofa a.s., Prague, Czech Republic), and tramadol (5 mg/kg; Tramadol-K, Krka d.d., Novo Mesto, Slovenia), a small incision was made above the spine through which the lower part of the belt punch pliers was slid beneath the skin. Consecutively, four round full-thickness excisions, 4 mm in diameter, were performed on the back of each rat. The incision was sutured after this procedure.

#### 2.1.2. Low-Level Laser Therapy

Three wounds on each rat were irradiated daily (for a maximum of six days) with a commercially available gallium-aluminum-arsenium (GaAlAs) diode laser (Maestro/C CM, Medicom Praha, Prague, Czech Republic; *λ* = 635 nm; oval shape of beam time of treatment at 15 mW/cm^2^ = 5 min 33 s, at 5 mW/cm^2^ = 16 min 40 s, and at 1 mW/cm^2^ = 83 min 20 s; probe distance to wound was 10 cm) to administer the total daily dose of 5 J/cm^2^, while the fourth wound was not irradiated and served as a control. One of the laser-treated wounds was irradiated at 1 mW/cm^2^ power density, the second at 5 mW/cm^2^, and the third one at 15 mW/cm^2^. The positions of the laser-treated and control wounds were rotated within the groups. During treatment, the rats were restrained in a Plexiglas cage with an oval opening over each currently stimulated wound, and the other wounds were protected from the reflected laser light.

#### 2.1.3. Histopathological Evaluation

Eight animals from each group were killed by ether inhalation two days, six days, or 14 days after surgery. The tissue specimens were processed routinely for light microscopy (fixation, dehydrating, embedding, and sectioning) and staining with hematoxylin and eosin (HE, basic staining) and van Gieson's stain (VG, nonspecific collagen staining).

A semiquantitative method was used to evaluate the following histological structures/processes, that is polymorphonuclear leukocytes (PMNLs), reepithelization, fibroblasts, new vessels, and collagen synthesis. The sections were evaluated in a blind manner, according to the scale 0, 1, 2, 3, and 4 (Table 1). To determine the inflammatory phase, we calculated the ratio of the number of PMNLs to the number of tissue macrophages (TMs) in specimens removed from animals killed two days after surgery. The numbers of PMNLs and TMs were counted in one high-resolution field from each section.

#### 2.1.4. Statistical Analysis

For each evaluated parameter mean values ± standard deviations (SDs) were calculated. The data obtained from the semiquantitative evaluation were compared using the nonparametric Kruskal-Wallis test. Significance was accepted at *P* < 0.05.

For the comparison of the PMNL/TM ratios, an analysis of variance (ANOVA) followed by Tukey-Kramer's multiple comparison test was used.

## 3. Results

**Figure 1:**
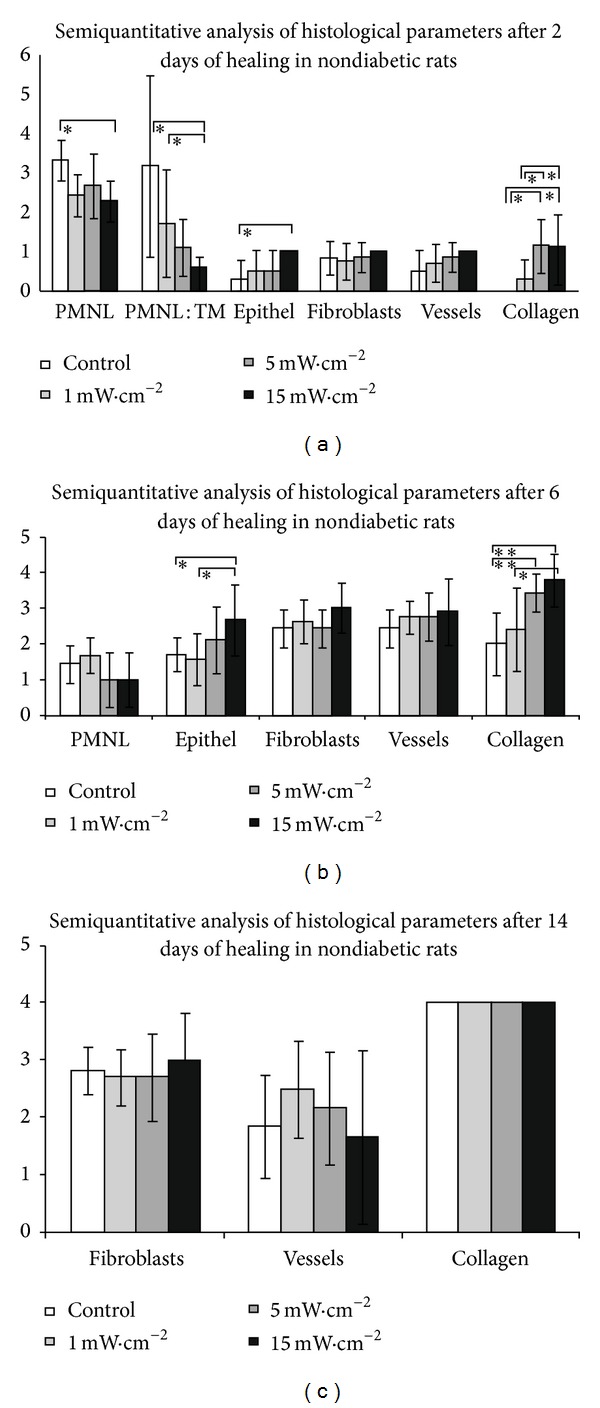
Results in nondiabetic rats group. (a) Results of the semiquantitative analysis of histological evaluation of healing skin wounds after 2 days of healing in nondiabetic rats group (data are presented as means ± SDs; *P < 0.05). (b) Results of thesemi-quantitative analysis of histological evaluation of healing skin wounds after 6 days of healing in non-diabetic rats group (data are presented as means ± SDs; *P < 0.05, **P < 0.01). (c) Results of thesemiquantitative analysis of histological evaluation of healing skin wounds after 14 days of healing in nondiabetic rats group. There was no significant difference between laser-treated groups and control group.

**Figure 2:**
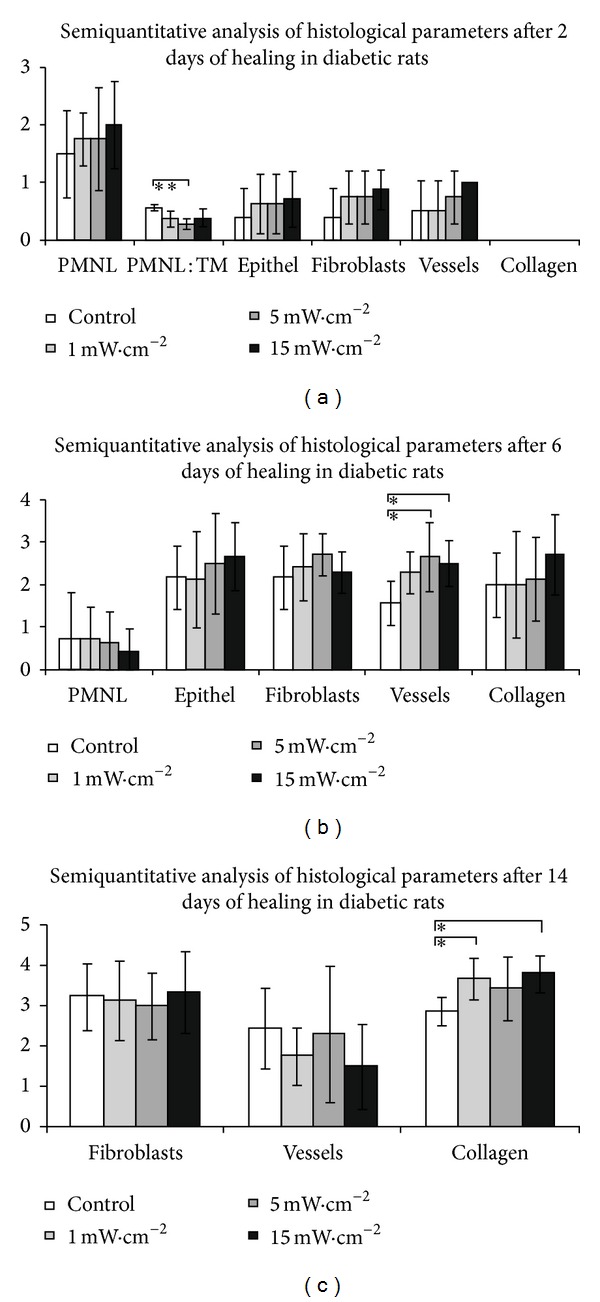
Results in diabetic rats group. (a) Results of the semiquantitative analysis of histological evaluation of healing skin wounds after 2 days of healing in group diabetic rats (data are presented as means ± SDs; **P < 0.01). (b) Results of thesemiquantitative analysis of histological evaluation of healing skin wounds after 6 days of healing in diabetic rats group (data are presented as means ± SDs; *P < 0.05). (c) Results of the semiquantitative analysis of histological evaluation of healing skin wounds after 14 days of healing in diabetic rats group (data are presented as means ± SDs; *P < 0.05).

**Figure 3:**
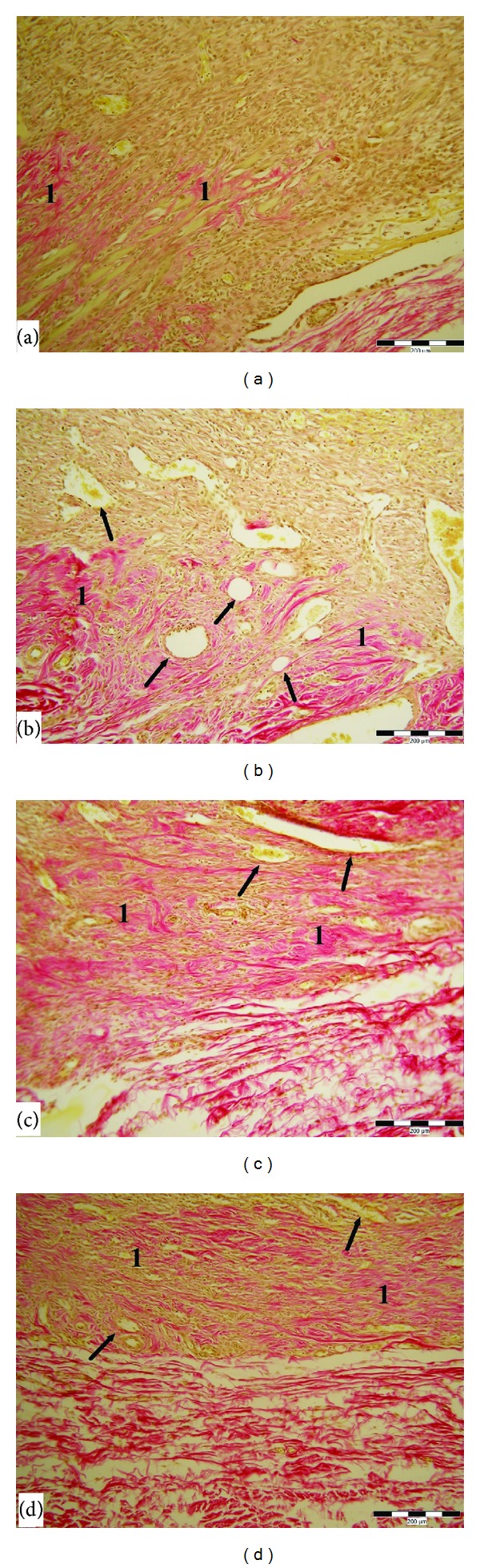
Skin wound after 6 days of healing in nondiabetic rats. (a) Control wound (1—granulation tissue contains a small amount of collagen). (b) Wound treated by power density, 1 mW/cm^2^ (1—granulation tissue with newly formed collagen, arrows marked new vessels). (c) Wound treated by 5 mW/cm^2^ (1—granulation tissue contains significant amount of new collagen, arrows marked new vessels). (d) Wound treated by 15 mW/cm^2^ (1—granulation tissue contains significantly the greatest amount of new collagen, arrows marked new vessels). VG stain; scale bar represents 200 *μ*m.

**Figure 4:**
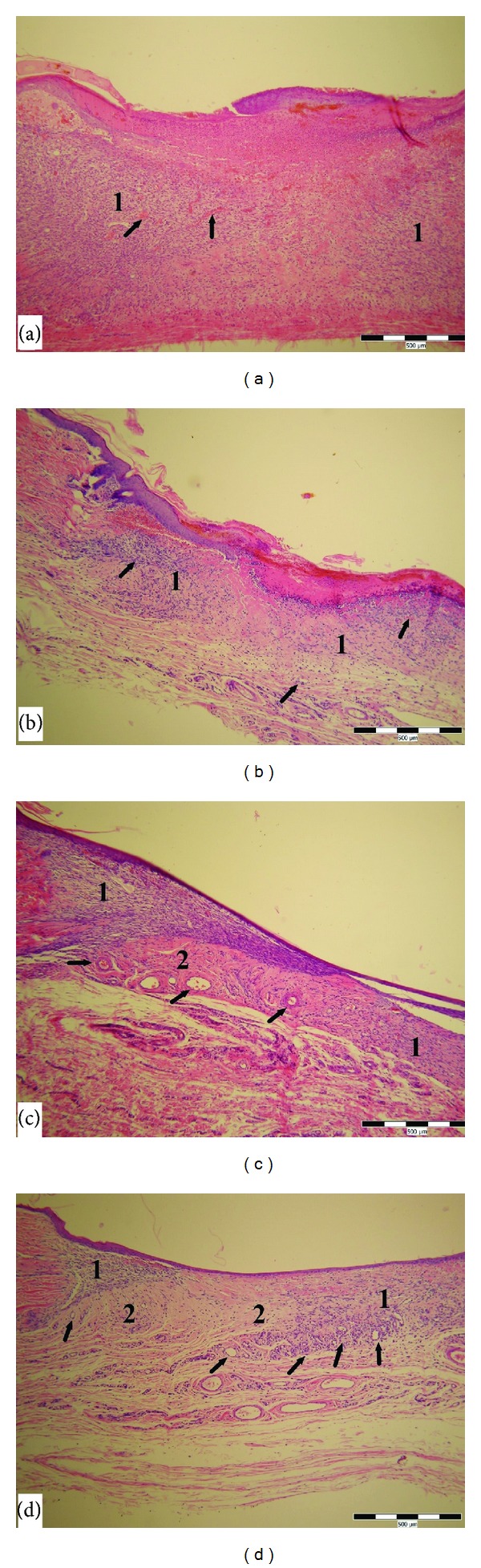
Skin wound after 6 days of healing in diabetic rats. (a) Control wound (1—granulation tissue of the wound, arrows marked isolated new vessels). (b) Wound treated at 1 mW/cm^2^ LLLT (1—granulation tissue of the wound, arrows marked proliferating fibroblasts and forming new vessels). (c) Wound treated at 5 mW/cm^2^ LLLT (1—proliferating fibroblasts, 2—granulation tissue rich in new collagen, arrows marked forming new vessels). (d) Wound treated at 15 mW/cm^2^ LLLT (1—proliferating fibroblasts, 2—granulation tissue rich in new collagen, arrows marked forming new vessels). HE stain; scale bar represents 500 *μ*m.

**Figure 5:**
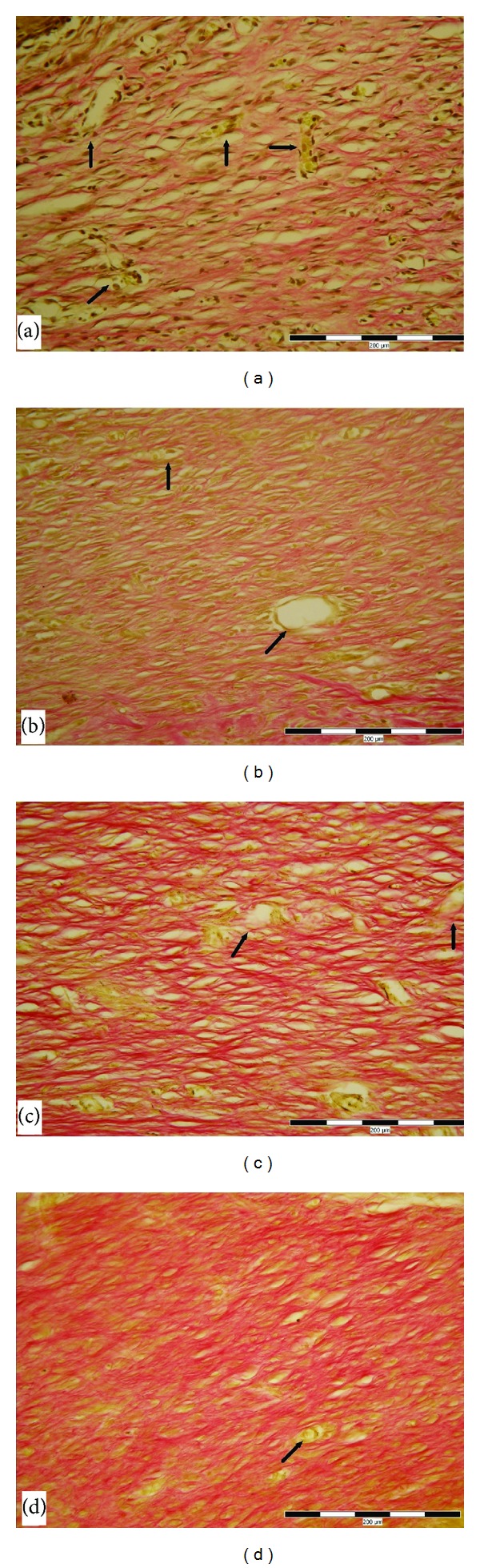
Skin wound after 14 days of healing in diabetic rats. (a) Control wound (granulation tissue of the wound; collagen fibers are colored in pink; arrows marked isolated new vessels). (b) Wound treated at 1 mW/cm^2^ LLLT (granulation tissue of the wound; collagen fibers are colored in pink; arrows marked isolated new vessels). (c) Wound treated at 5 mW/cm^2^ LLLT (granulation tissue of the wound rich in new collagen, colored in pink, arrows marked only isolated new vessels in regression). (d) Wound treated at 15 mW/cm^2^ LLLT (granulation tissue of the wound with a significant amount of new collagen, arrows marked new vessels in regression). VG stain; scale bar represents 200 *μ*m.

During the post-surgery period, the animals remained healthy, with no clinical evidence of infection. The results of our investigation are summarised in Figures 1–5.

### 3.1. Nondiabetic Group

#### 3.1.1. Two Days after Surgery

PMNL infiltration was found to be significantly lower in the irradiated group with 15 mW/cm^2^ of power density (*P* < 0.05) when compared to the control group. The ratio of PMNL/macrophages was significantly higher in the control group and in wounds irradiated by 1 mW/cm^2^ compared to wounds irradiated by 15 mW/cm^2^  (*P* < 0.05) (Figure 1(a)). The layer of epithelium was observed to be thickened at the edge of the wound after 48 hours in the control group and irradiated groups (1 mW/cm^2^ and 5 mW/cm^2^), while the start of keratinocyte migration to the middle part of the wound in the 15 mW/cm^2^group was observed. A significant difference (*P* < 0.05) in the amount of newly formed collagen in the irradiated group (5 mW/cm^2^ and 15 mW/cm^2^) was recorded compared to 1 mW/cm^2^ irradiation and control wounds (Figure 1(a)).

#### 3.1.2. Six Days after Surgery

Acute inflammation phase of wound healing was completely finished; there was only accidental infiltration of a few polymorphonuclear leukocytes in all irradiated and nonirradiated wounds without statistical relevance after six days of treatment. Histologic examination after six days was revealed on the surface of excisions the formation of an almost continual layer in the 15 mW/cm^2^ group with a significant difference compared to the control and 1 mW/cm^2^ groups. Proliferation, migration of fibroblast, and neoangiogenesis was slightly accelerated in the 1 mW/cm^2^ group, but without significant differences compared to the 1 mW/cm^2^, 5 mW/cm^2^, and controls. The synthesis and organisation of collagen fibres were significantly enhanced in the 5 mW/cm^2^ and 15 mW/cm^2^ groups compared to the others. (Figures 1(b) and 3).

#### 3.1.3. Fourteen Days after Surgery

The changes in the wound healing of the skin were not any more remarkable in the later phase of maturation; even the effect of LLLT was without a statistical significance (Figure 1(c)). Healing remodeling and reorganisation of extracellular matrix (ECM) were characterised.

### 3.2. Diabetic Group

#### 3.2.1. Two Days after Surgery

Formation of the demarcation line beneath the scab consisting mainly of polymorphonuclear leukocytes was seen mostly in wounds which received a laser beam with a power density of 15 mW/cm^2^, but there was no significant difference between each group. Epithelization, fibroplasia, and neoangiogenesis were only slightly enhanced in all stimulated wounds, without significant differences. The only significant difference was recorded in the ratio PMNL/Ma in wounds stimulated by 5 mW/cm^2^ compared to the others (*P* < 0.01) after two days of irradiation (Figure 2(a)).

#### 3.2.2. Six Days after Surgery

The wound healing process in diabetic rats was delayed. A significant difference in the number of newly formed capillaries in the irradiated groups (5, 15 mW/cm^2^) was shifted to later phase (six days after injury) compared to the control group (Figures 2(b), 4(c), and 4(d)). There was no statistical significant difference between the control and irradiated groups in the other parameters.

#### 3.2.3. Fourteen Days after Surgery

With regard to the effect of LLLT on wound healing in diabetic rats, the changes in the formation of collagen fibres were substantially more remarkable in the later phase of wound healing. The effects of LLLT on the quantity of collagen were seen after 14 days of healing in the diabetic rats, and the statistically significant difference was evaluated in 1 and 15 mW/cm^2^ compared to the control group (Figures 2(c) and 5).

## 4. Discussion

LLLT as a therapeutic modality was introduced by the work of Mester and colleagues, who noted an improvement in wound healing with the application of a low-energy (1 J/cm^2^) ruby laser [13, 15]. LLLT has been used clinically to stimulate the healing of a variety of musculoskeletal injuries such as tendinitis and soft tissue injuries, as well as open skin wounds, and in the treatment of various skin conditions such as psoriasis and acne [12, 13]. Despite the widespread use of LLLT in the treatment of wound healing, the exact mechanism of action of LLLT is not yet known. It was found that cytochrome c oxidase becomes more oxidized due to the irradiation at each of the wavelengths used [19]. It can be hypothesized that the mechanism of LLLT at the cellular level is based on an increase in the oxidative metabolism in the mitochondria [19, 20]. Laser treatment is characterised by a number of physical parameters such as wavelength, spot size, power, power density, energy, energy density, and duration of irradiation. However, at present, the relevance of these parameters to the healing effects of laser irradiation on different injuries and skin conditions remains unclear.

Considerable variation has been identified in the used wound models and in laser parameters used in reviewed studies. Because of this, the direct comparison between studies and the establishment of optimal irradiation parameters for LLLT, such as recommended dosages, wavelength, and power density, is not yet possible [12]. It has been documented in numerous studies that LLLT positively influences the wound healing process by accelerating inflammation, promoting fibroblast proliferation and neoangiogenesis, facilitating collagen synthesis, and reducing postoperative pain [21–24]. Nevertheless, several researchers have shown that LLLT may have adverse effects on wound healing or that there has been no positive effect of LLLT on the wound healing process [16, 17, 25]. These contradictory results can be a negative consequence of such a wide variability of used laser parameters in individual studies. The aim of our study was to compare the effects of different power densities with equal daily doses of LLLT at 635 nm on the healing of excisional skin wounds in nondiabetic and diabetic rats in order to optimise the parameters of LLLT. He-Ne laser (632.8 nm) belongs to the most common devices used in LLLT, so we decided to test diode laser which produced radiation at 635 nm, comparable to He-Ne laser radiation. In terms of tested power densities (1, 5, and 15 mW/cm^2^), we have found better results using higher power densities. Laser irradiation with higher power densities accelerated inflammation, epithelization, neoangiogenesis, and collagen production in our current study. This finding is in agreement with the results of a study published by do Nascimento et al., in which LLLT was found to be more effective with the higher intensity combined with the shorter wavelength or lower intensity with a higher wavelength [26]. In our previous works looking at the influence of different power densities of LLLT at 635 nm and 670 nm on wound healing in normal and corticosteroid-treated rats we demonstrated that LLLT at 635 nm and 670 nm effectively stimulates wound healing by using higher power densities [14, 27]. Histological analysis correlated with significantly increased wound tensile strength at 635 nm in a power density-dependent manner, whereas LLLT at 670 nm increased wound tensile strength by using a lower power density [28]. According to our previous study, LLLT has a protective effect against early inflammatory tissue response after ischemic reperfusion injury of muscle, with a high power density of 40 mW/cm^2^ [29].

In our study we used a daily dose of laser irradiation of 5 J/cm^2^ as a comparison of the effects of laser therapy in other studies with different doses and wavelengths, where a median dosage of 4.2 J/cm^2^ was recorded [12]. It has been well documented that the He-Ne laser, using a dose of 4 J/cm^2^or 5 J/cm^2^, accelerates wound closure, increases collagen deposition, and has a stimulatory influence on wounded fibroblasts [23, 30, 31]. Yasukawa and coworkers showed that LLLT with He-Ne laser applied as a daily dose of 4.21 J/cm^2^ significantly increased the tensile strength and inhibited inflammation and increased formation of collagen fibres and recovery in the continuity of tissues, compared to a daily dose 2.09 J/cm^2^ [32]. A similar finding was reported in the work of Stadler et al. who investigated the effect of laser irradiation at 830 nm and a daily dose of 5 J/cm^2^ on incisional wound healing in diabetic mice. LLLT with these parameters significantly enhances cutaneous wound tensile strength in this study. Stadler et al. used an equal daily dose as in our current study [33]. Yu et al. have shown that treatment with a 630 nm Argon dye laser at a fluence of 5 J/cm^2^ enhanced the percentage of wound closure over time as compared to the negative control group in genetically diabetic mice [34]. In our previous studies we confirmed a positive effect of He-Ne, GaAlAs, and AlGaInP laser irradiation at a dose of 3 J/cm^2^ and 5 J/cm^2^ on primary and secondary wound healing [14, 27, 28, 33, 35]. In contrast, inhibition of the wound contraction under He-Ne and Argon laser exposure to 20 J/cm^2^ was recorded [23], a smaller expression of collagen and elastic fibres with a dosage of 8 J/cm² compared with 4 J/cm^2^[36], and a decrease in cell viability and cell proliferation of wounded fibroblast exposed to 10 and 16 J/cm^2^ compared with 5 J/cm^2^ [31].

According to the present study, LLLT accelerated the inflammatory phase in nondiabetic animals with more significant difference by 15 mW/cm^2^ power density. The anti-inflammatory effect of laser radiation in diabetic animals was observed by the power density-dependent decrease in PMNL/TM ratio with the best results using power density 5 mW/cm^2^. Many researchers confirmed the anti-inflammatory effect of laser radiation [5, 6, 22, 33, 37, 38]. By contrast, in the group of diabetic rats an increased wound infiltration by inflammatory cells with less effectivity of LLLT was observed. Deceleration of the inflammatory phase is a negative consequence of diabetes on wound healing. The most probable explanation for this observation is that diabetes strongly decelerates the inflammatory phase compared to nondiabetic rats, while the effect of laser irradiation is not able to sufficiently improve the metabolic demand and affected nutrition in the first days of application. We have no data at our disposal to support this.

We observed that LLLT positively influences the proliferative phase of wound healing with significant acceleration of epithelization and collagen synthesis in nondiabetic rats and neoangiogenesis in diabetic rats. The literature documents many studies about acceleration of epithelization, fibroblast migration and proliferation, collagen synthesis, and neoangiogenesis during LLLT [12, 13, 23, 30, 39]. Reddy et al. and Maiya et al. [22, 37] showed that laser photostimulation accelerates collagen production in healing wounds of diabetic rats. In addition, Maiya et al. showed in their study a significant increase in fibroblastic proliferation, capillary proliferation, granulation tissue formation, and epithelization in the He-Ne laser-treated group of diabetic rats as compared to the control group of diabetic rats. These results were consistent with our results. Stadler et al. examined the effect of laser irradiation at 830 nm with a fluence of 5 J/cm^2^ in a murine diabetic model. It was determined that laser irradiation significantly enhances cutaneous wound tensile strength in this murine diabetic model [40]. This result may be related to the finding of accelerated production of collagen in wounds treated with LLLT. Speeding up the proliferative phase in the process of healing in both groups of rats was reported in a power density-dependent manner, with better results found by using higher power densities—5 and 15 mW/cm^2^. Better results are recorded in the group of nondiabetic rats. Delayed wound healing in diabetes might be responsible for this decreased effectivity of LLLT.

With respect to the maturation phase of wound healing, it was found in our study in the group of nondiabetic rats that there was no significant difference between the irradiated and control wounds. On the other hand, in the group of diabetic rats we observed a significantly greater amount of collagen in the wounds treated with LLLT. Our finding is consistent with the arguments of several researchers [22, 37].

In conclusion, in our study LLLT has a positive effect on the wound healing process in both groups, nondiabetic and diabetic rats. The results of the present study show that LLLT at 635 nm has an anti-inflammatory effect at 5 mW/cm^2^ and 15 mW/cm^2^ power densities. Laser irradiation enhances recovery after secondary skin injury in diabetic rats as well as in the nondiabetic group by facilitating collagen production and neoangiogenesis at 5 mW/cm^2^ and 15 mW/cm^2^ power densities. Maturation phase is enhanced by LLLT only in the group of diabetic rats. Overall, by using LLLT in a diabetic rat model of excision skin wounds, the present study suggests that a therapeutic dosage of 635 nm and 15 mW/cm^2^ may promote skin wound healing.

## Figures and Tables

**Table 1:** Semiquantitative histological evaluation of healing skin wounds.

Scale	Reepithelization stage	Polymorphonuclear leukocytes	Fibroblasts	New vessels	Collagen
0	Thickening of cut edges	Absent	Absent	Absent	Absent-granulation tissue
1	Migration of cells (<50%)	Mild-surrounding tissue	Mild-surrounding tissue	Mild-surrounding tissue	Minimal-granulation tissue
2	Migration of cells (≥50%)	Mild-granulation tissue	Mild-granulation tissue	Mild-granulation tissue	Mild-granulation tissue
3	Bridging the excision	Moderate-granulation tissue	Moderate-granulation tissue	Moderate-granulation tissue	Moderate-granulation tissue
4	Keratinization	Marked-granulation tissue	Marked-granulation tissue	Marked-granulation tissue	Marked-granulation tissue
